# Supplementary figures and images for: “I want to perform and succeed more than those who are HIV-seronegative” Lived experiences of youth who acquired HIV perinetally and attend Zewditu Memorial Hospital ART clinic, Addis Ababa, Ethiopia
Source: PLoS One. 2021 May 27;16(5):e0251848. doi: 10.1371/journal.pone.0251848 (PMC8158987; doi:10.1371/journal.pone.0251848)

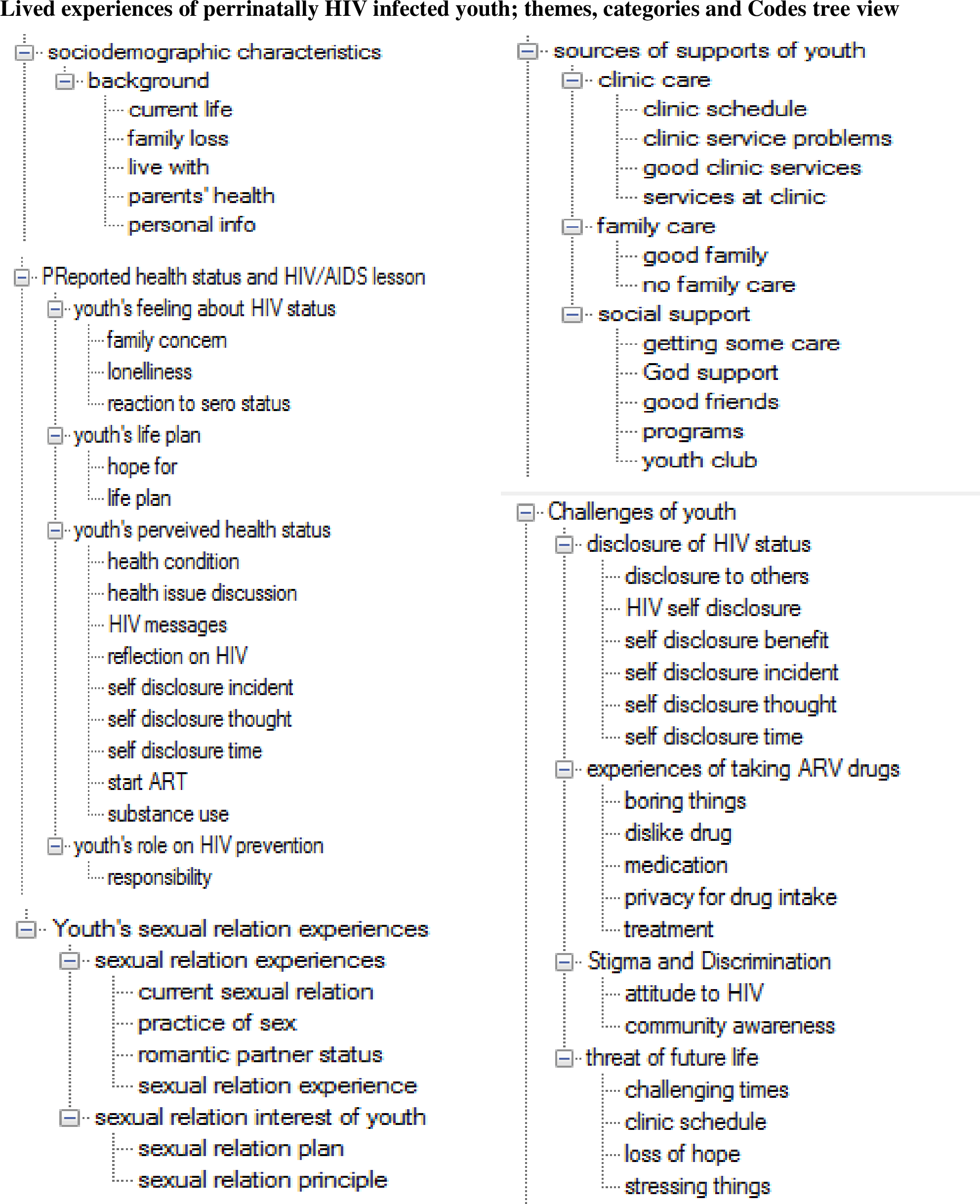

Supplement: S1 Fig — (TIF) [file pone.0251848.s001.tif]
