# Supplementary material for: “I want to perform and succeed more than those who are HIV-seronegative” Lived experiences of youth who acquired HIV perinetally and attend Zewditu Memorial Hospital ART clinic, Addis Ababa, Ethiopia
Source: PLoS One. 2021 May 27;16(5):e0251848. doi: 10.1371/journal.pone.0251848 (PMC8158987; doi:10.1371/journal.pone.0251848)
Supplement: S1 Table — (DOCX) [file pone.0251848.s002.docx]

**S1 Table.**

**Annex-VI: Lived experiences of perrinatally HIV infected youth, 2018; Code book**

| code | description | example |
| --- | --- | --- |
| Personal info | Description of age, sex, education, occupation, marital status | If one says ‘ I am a student’ |
| Parents’ health | Mother and father’s health | If said ‘my mother has died’ |
| Live with | Description of living condition how and with whom one lives | If said ‘I live with aunt’ |
| Health feeling | Any description of general health status | If one says ‘I am healthy’ |
| HIV self disclosure | Description of how and when they knew their status | If said ‘I knew my status incidentally’ |
| Time of disclosure | Description of timing in disclosing HIV status to self and others | If one says ‘ it will be good if children know their status from childhood |
| Reaction to sero status | Description of any reaction to knowing their sero status | If one said ‘ I cried when told me as I have HIV |
| Experience of drug intake | Description of taking ARV drug | If one says ‘ I have being taking ARV drug since my childhood’ |
| Good family | Description of any care from families | If one says ‘I have a family who supports me….’ |
| Clinicians’ care | Description of approaches and services from health care providers | If one says “the care providers have a well coming face and are kind….” |
| Clinic services | Any clinic services in terms of access, quality, and adequacy | If said as “I can get the service I need any time…” |
| Social support | Any positive approach and support from friends, neighbors, teachers,…. | If said as “my friends are like my family, they understand my feeling and support me in many things…” |
| Peer group | Description of any groups found and any experience related with | If said ‘we have a group of HIV positive youths |
|  |  |  |
| Fear of stigma | Any description of thinking about stigma from different sides | If said as “I can’t take my drugs if people are around me….” |
| Attitude to HIV | Description of any attitude to HIV | If said ‘community’s attitude to HIV positives is not good’ |
| Changing things | Description of suggested things that needs improvement | If said ‘ I wish if community’s awareness is changed’ |
| Reaction to HIV messages | Description of reactions to HIV messages from different medias | If said ‘I feel nothing for HIV messages’ |
| responsibility | Description of role and responsibilities in HIV prevention | If one says ‘we should take care of others’ |
|  |  |  |
| Drug intake | Any description related with drug’s taste, load, timing…. | If said the drug has bad taste…. |
| Family concern | Any description of thinking about missing, having and planning to form a family | If said as “I wish to have a family….” |
| self disclosure thought | Any description of disclosing or not of self status | If said as “I wish to disclose my status but I fear….” |
| loneliness | Any description of feeling loneliness and why | If said as “I usually stay alone…” |
| Stressful things | Description of any stressful conditions for them | If said ‘I worries if I fails in my education’ |
| threats | Description of any threatening things | If said ‘my threat is if getting job needs medical certificate’ |
| Family loss | Any family loss and challenges related with | If said “I missed family member |
| Economical issues | Any description of income, food and other insecurity | If said as “I can’t get enough money to buy food….” |
| Clinic schedule | Any description related with clinic appointment timing vs other personal deeds inconvenience | If said as “I miss class not to miss my clinic appointment…” |
|  |  |  |
| Disclosure to others | Any description of challenges related with not disclosing self status | If said as “I may not get other medical services at other clinics with out disclosing my status |
|  |  |  |
| reproductive health | Any description of reproductive health issue | If said “I am not familiar with the issue of reproductive health…” |
| Past Sexual relation | Any description of experiences related to sex | If said “I had had a friend and we had sex with condom |
| Current Sexual relation | Any description of current sexual relation status | If said “I have no sexual experience now…” |
| sexual relation plan | Description of what is the thought and plan of sexual relation | If said as “I wish to have a boyfriend…..” |
| sexual relation Principle | Description of what the sexual relation should be | If said as “I believe we should be care full of not to infect others…” |
| life plan | Description of future plan in life | If one says ‘I want to be doctor’ |
| Things to do | Description of what they wish to be done for them | If said ‘ I wish to eradicate HIV’ |
| health issue discussion | Description of any discussion about self health condition with any one | If said ‘ I talk with my friends about my health’ |
| Boring things | Description of any things that are boring | If said ‘ I am boredom of taking drugs’ |
| Loss of hope | Description of any things that reveal loss of hope | If said ‘ I have no hope’ |
