# Supplementary material for: “I want to perform and succeed more than those who are HIV-seronegative” Lived experiences of youth who acquired HIV perinetally and attend Zewditu Memorial Hospital ART clinic, Addis Ababa, Ethiopia
Source: PLoS One. 2021 May 27;16(5):e0251848. doi: 10.1371/journal.pone.0251848 (PMC8158987; doi:10.1371/journal.pone.0251848)
Supplement: S1 Data — (ZIP) [file pone.0251848.s003.zip › S1_Data/3M18-03 word.docx]

**Study Title: Lived Experiences of Perinatally HIV Infected Youths**

**Unique ID**: M18-03

**Interview category**: In depth interview

**Interviewer name**: Nahom

**Interview date**:

**Interview duration**: 34’:13’’

**Place:** Addis Ababa

**Transcriber name**:Nahom

Section1:- Socio demographic characteristics

I: how old are you?

R: my age is 18

I: ok your educational status

R: I am grade 10 student

Sex: Male

Religion: Protestant

Marital status: single

Occupation: student

I: with whom are you living?

R: I live with my aunts

I: ok your aunts, are they that of mother’s or father’s?

R: mother’s sisters

I: ok what do you know about your father and mother?

R: my father, my father died, he had had the virus and I also got it from him, my mother also got from him because she was healthy and her sisters are also healthy. He died when my mother was pregnant. My father assigned my name and she also died after some periods when I was around 4 or 5 years old, she died due to illness.

I: but have they told you as she died from that (to mean HIV)?

R: yes

I: from HIV?

R: yeaha

I: ok since that time, are you living with your aunts?

R: yeaha

**Section 2:-health status**

I: how is your health as general? That means your general health beyond your HIV status

R: I feel as I haven’t have hope

I: why don’t you have?

R: I don’t know

I: beyond that (to mean beyond HIV), have you ever had any illness?

R: no, I didn’t have

I: any type of illness?

R: yeaha

I: when have you known as you have it(HIV)?

R: it is around when I was around 6 or 7 years old, it was my aunt who took me to clinic when I was sick and she knew it as I have. By that time my aunt first refused for me not to take drug but after much argument she accepted and I started to take it (the ARV drug).

I: have you known by that time?

R: yeaha, I started in that way; by that time when you take it, it vomits you and when it vomits me I used to discontinue.

I: have you known it by that time?

R: but she reminds me

I: But have you known as you have HIV?

R: no I haven’t known

I: ok when was? When have you known as you have HIV? When you knew it well?

R: she brought me for taking drug and the doctors used to advise me as I should limit myself from playing much as that of my peers because they said me “you are not as others, you should not expend much energy”

I: have they said that?

R: yeaha

I: when have you known well as you have it?

R: I don’t know

I: don’t you remember it?

R: yeaha I don’t remember

I: ok but since when have you known it well?

R: it is around when I was grade 7

I: ok you knew it well when you were grade 7? What have you felt by that time?

R: since I adapted it I felt nothing

I: ok nothing special happened? You felt no special thing?

R: but now being not equal with others created on me a bad feeling. I took the drug 1 at morning and 1 at night, so sometimes when there is a program…..

I: what program

R: with friends

I: ok, since you should take on time?

R: yeaha I may not finish with them, I should go to home

I: you knew your status well when you was around grade 7(that means around age 15), but for others like you, when do you think is better to know it? When should they know?

R: It may have a challenge if they knew at early age

I: so which age is better?

R: around 14 or 15 years old

I: ok you think it is good if it is in that way?

R: yeaha

I: what do you think are supportive things for you?

R: good thing?

I: yeaha good things; for example let start from family side, from family side what are the things you take as supportive for you?

R: for me all is good

I: ok what from others side; like school?

R: from school, no one knows

I: your teachers also don’t know?

R: they don’t know, since they are changed every time I didn’t tell to any one

I: ok what about from clinic side?

R: clinic? The same but I come for appointment missing class, even for CD4 checkup I should come at a very early in the morning?

I: is early a must?

R: yeaha, there is queue, even now it is relatively better, I used to come at 4:00 a.m.

I: can’t you come on Saturday for your clinical appointment?

R: Saturday, I can but even Saturday we have class, even it is hard on that day because we have many classes

I: ok what do you say for teachers when you miss the class?

R: I have this permission (showing medical certificate)

I: but it doesn’t reveal as you have HIV?

R: yeaha it doesn’t

I: do you fear when you come here? Do you fear if anyone knows your status?

R: no I don’t fear

I: do your friends know your status?

R: no

I: who knows about you?

R: about me relatives know, but no stigma and the like.

I: nothing you felt such things?

R: yeaha, I haven’t felt

I: what do you feel when people talk about HIV in your school?

R: mmmm people see it as joke, when they joke you also joke with them

I: but have you ever faced when HIV is described as bad or good?

R: since I don’t give attention, there is no

I: for example how do your friends describe a person with HIV?

R: since I haven’t ever asked them, beyond education I don’t need to talk about it

I: why you don’t like to talk?

R: I don’t know, but I don’t like it

I: what are the things you are concerned of? What do you think are concerning issues for you?

R: if I don’t succeed with my education what may I do for future? This concerns me

I: what else?

R: it is that what may I do for future if I don’t succeed? I heard there is drug for six month but it didn’t come still

I: who said you?

R: I heard as there is such drug

I: a rumor?

R: yeaha a rumor

I: what else about life? like with respect to getting what you need?

R: as my family, I get what is there in the family

I: do you have any income by yourself?

R: no I don’t have

I: have you ever faced any serious health problem?

R: no

I: what things you think are good for you if they are done?

R: if the drug for six month comes

I: what else?

R: nothing

I: Are there things you think are challenging for you?

R: if the drug, treatment doesn’t come living in this case….(silence)

I: now you are saying the drug is difficult, relative to timing is not comfortable

R: yeaha, if I started job how I may….always drug, it is boring

I: what else? You said me only your family know your status, do you take your drugs freely if you are with other people in your drug taking time?

R: no, I take it in separate class being alone; I don’t take while they see me

I: now only your aunts know?

R: no, my families even from countryside know, since they regret much about my mother’s death and they take it that lesson.

I: do your cousins know?

R: yeaha

I: What do they say you?

R: nothing, they don’t give it a place

I: what cares do you take?

R: around the beginning they told me to take care of blood, so I also took care of

I: but do you play with them heartily as you need? You eat with them; No problem?

R: yeaha, yeaha

I: in school also? No stigma and the like?

R: yeaha,

I: ok is there anything you heard of about relationship?

R: relationship

I: relationship means with respect to opposite sex relationship

R: nothing

I: here in the hospital also? Don’t they (health care providers) talk you about?

R: yeaha

I: here (the hospital) isn’t there a program in which you get together?

R: youth?

I: yeaha, is there by now? Every what time do you meet?

R: every 15 days

I: is there by now?

R: yes

I: do you meet well? With your age group and those who are above you?

R: yeaha

I: are there males and females?

R: yeaha

I: what do you talk when you meet?

R: when we meet mmmm we play, we learn, we talk about opposite sex relation and also about our drug.

I: you talk with males and females in a group?

R: yes

I: what do you talk about opposite sex relation?

R: opposite sex, it is about when we should have a friend (opposite sex relation)

I: ok now you, when do you think you may have a girl friend?

R: I don’t give it place

I: why?

R: I think I am yet?(gena negn biye asbalehu)

I: but in your future like in your 20s and above age, what type of girlfriend you think to have?

R: I think that if I have enough income

I: ok if you have that also, what type of friend you wish to have?

R: if I get a person like me (to mean HIV positive woman)

I: what about beyond that?(to mean HIV negatives)

R: I don’t think

I: don’t you think?

R: Yeaha

I: Why?

R: I don’t like to hurt others

I: ok in your group discussion what do your friends say about this?

R: I started it recently; we didn’t talk about it that much

I: do you have friends in school?

R: I have but I don’t have that much close friends

I: why don’t you have close friends?

R: I don’t give attention that much

I: what is your future plan in your education?

R: I want to be a pilot

I: ok what other things you like if it is done for you?

R: if our youth program is done in every week, again we are classified in to two and don’t have enough class, and if other program is added in which we play freely like tour is prepared for us

I: but are there many people who come?

R: yes

I: how you classified?

R: below 16 years and above

I: ok what other things you would like to say

R: I wish if eradicating thing is gotten or if not gotten I wish at least the six month’s drug comes (matfiyaw bigegniln weyim kaltegegne deemo ye sidst woru medhanit bigegniln)

I: have you ever faced news of who said cured from HIV?

R: yeaha I heard as it gets cure in religion

I: so have you ever tried any?

R: yes, praying was done for me

I: ok I have finished thank you! If I missed anything I may revisit you as per your willingness

R: ok
