# Supplementary material for: “I want to perform and succeed more than those who are HIV-seronegative” Lived experiences of youth who acquired HIV perinetally and attend Zewditu Memorial Hospital ART clinic, Addis Ababa, Ethiopia
Source: PLoS One. 2021 May 27;16(5):e0251848. doi: 10.1371/journal.pone.0251848 (PMC8158987; doi:10.1371/journal.pone.0251848)
Supplement: S1 Data — (ZIP) [file pone.0251848.s003.zip › S1_Data/4M19-04 word.docx]

**Study Title: Lived Experiences of Perinatally HIV Infected Youths**

**Unique ID**: M19-04

**Interview category**: In depth interview

**Interviewer name**: Nahom

**Interview date**:

**Interview duration**: 34’:57’’

**Place:** Addis Ababa

**Transcriber name**:Nahom

Section1:- Socio demographic characteristics

I: you said me your age is?

R: 19

I: ok your education level

R: I am grade 8 student

I: grade eight ok

Sex: Male

Religion: Orthodox

Marital status: single

Occupation: student

I: with whom are you living?

R: with my mother

I: with your mother okay, what about your father?

R: my father has died

I: when?

R: when I was 7 years old child

I: when you were 7 years old child? Aha aha ,ok do you have sisters and brothers?

R: no I don’t have

I: aha aha you are the only son, how is your mother?

R: she is well

**Section 2:- Health status**

I: as a general what do you feel of your health condition? Beyond HIV issues how is your general health? What do you feel of it?

R: I feel healthy and I think as I can perform any thing as that of any person who is healthy

I: have you ever faced any other disease?

R: no I haven’t, but when I was kid I had had wart on my skin and kidney problem but I went to spiritual water then after I am healthy (tsebel hedkugn, kezia danku)

I: ok when did you know as you have HIV?

R: when I was around 9 or 10 years old

I: aha aha how did you know? How was the incident?

R: it is my mam who told me

I: your mother, how she told you? Was it at home or health center?

R: health center

I: what she said you?

R: when I said her why ‘I come here to clinic every time’, she said me that “we are not as other people, we have HIV, so we come here to clinic to take treatment”

I: aha aha, you started the drug before you knew that you have HIV?

R: yeaha

I: when you started to take the drug? At what age have you started?

R: I don’t remember

I: but it is before your 9 years of age, so you asked why you come to clinic?

R: yeaha

I: ok so your mother told you that, ok what about physicians?

R: they also advised me not to stress

I: ok what have you felt when your mother told you?

R: since I was kid I don’t remember it

I: aha aha what was your feeling when you become growing? When have you started to know about HIV in detail?

R: it is since around my 13 or 14 years of age

I: ok by that time when you became aware of HIV and your status what have you felt?

R: I knew that a person with HIV can also perform and live as any healthy person.

I: ok but that is from what people told you but what was your internal feeling about being HIV positive

R: nothing

I: you didn’t feel any special thing?

R: yeaha

**Section 3:- supportive things**

I: what are the things around you, you take as good for you? Let start from family side

R: it is only my mother I say good for me

I: what else? Let take from school side, what things are good for you?

R: I have one best friend

I: does he know your status?

R: yes

I: does he also have?

R: no

I: do teachers know your status?

R: no only my friend knows?

I: ok what else? What about from clinic side? Are there any things you say as good things for you?

R: all is good; they don’t see as a patient, they advise us

I: ok what else? Anything you have seen as good for you?

R: nothing I saw special

I: what other things you want to be done for you which you consider as good for you?

R: nothing

I: there is no that much?

R: yes

I: with whom do you discuss about your health?

R: only with my mother

I: aha only with your mother? Ok apart physicians no one knows about your status?

R: yeaha only my mother and my friend

I: ok don’t your neighbors know?

R: few of them know but they see us as healthy person

I: but you haven’t faced any negative views from them?

R: yeaha

I: what do you think of about telling to others?

R: it has no problem for me if I disclose for others

I: aha aha when do you think is good for you to know about your status?

R: any time it has no problem

I: for example for other children like you when do you think is the right time to tell them as they have HIV? You think is that when they are kid or when they grew a bit?

R: it is better when they grew

I: why

R: to have knowledge it is better to tell them when they grew, on that age they may have knowledge about HIV and all things so it is better if they are told when they grew a bit

**Section 4:- concerns**

I: are there any things that concerns you related to your health?

R: there is no that concerns me

I: nothing? Related to family and others?

R: there is nothing that concerns me

I: relative to messages about HIV from radio, posters and others side; is there anything you saw and felt about your status?

R: I don’t feel that much

I: is there a message you saw and said if it was in this way or in that way?

R: there is no

I: don’t you fear to disclose your status to any one?

R: yeaha

I: anywhere? For example do your friends talk about HIV in school?

R: they don’t talk but I know and see each other as healthy person

I: ok what do you think is your role for HIV not to be transmitted?

R: taking care

I: your role, when you say care? What type of care do you think you should do?

R: ….(silence)

I: don’t you learn?

I: we learn

I: ok so what type of care?

I: like using sharp material privately, use tooth brush privately

**Section 5:- challenges**

I: what challenges have you ever faced and things you take as challenges by now?

R: no challenging things

I: from family or school and others, no challenges?

R: yeaha

I: for example you came here to day here, so doesn’t it has a problem with your class? What do you say to teachers?

R: I told them as I went to clinic

I: do they ask you what type of illness?

R: no

I: you take medical certificate?

R: yeaha

I: but does the clinic appointment have effect on your education?

R: no

I: every what time do you come?

R: every one or two month

I: so you come for medical appointment by missing class?

R: yeaha

I: why don’t you come Saturday?

R: I also come Saturday, for example if I have drugs for two extra days from my appointment day I will come on Saturday or other days convenient for me

I: aha aha without affecting your class?

R: yeaha

I: how many times do you take your drug in a day?

R: morning and time

I: ok can you take your drug everywhere, like in school freely?

R: no, I hide

I: why?

R: I don’t want to hide but sometimes in bus

I: what do you do? So how you take your drug where there are people

R: I take it hiding from others, for example in a bus I turn myself to window cover myself with bag and take my drug

I: what enforced you to do that? Is that you think that ‘if they know they may stigmatize me?’

R: no my mother usually says me ‘people shouldn’t see’

I: why she says?

R: I don’t know

I: you know there are natural changes that come with age increment so what do you know about reproductive health? Tell me freely as talking with you friend

R: …..silence

I: anything you know, anything you heard from school or do they tell you here at clinic?

R: sometimes they told us

I: what things you have heard?

R: ………silence

I: tell me anything you heard and you feel? There is a program you come together in the clinic, right?

R: yeaha, it was before but I left it

I: why you left it?

R: they said elders above 18 years should leave

I: who did that? It is two groups; for children and elders, so why don’t you enter to elders group?

R: I enter but..

I: so what have you seen? What is there?

R: there, it is pleasant

I: ok tell me what are there? Tell me those pleasant things

R: all are the same group, they are pleasant, and since all are the same there is no fear.

I: aha aha you talk freely?

R: yeaha

I: about what do you talk?

R: about HIV and cancer diseases, as HIV is not very bad and others things

I: what do you think about having friend and others?

R: …..(silence)

I: tell me don’t hide me, you know it is a natural feeling for every one that comes at its time, so it is about how we respond for that feeling. So what is said about relationship?

R: …..(silence)

I: unless you fear to tell me there is something you know, ok when you meet with your friends there are males and females right? So don’t you talk about such things? Don’t you have friends who have relationship?

R: I don’t know

I: ok what do you feel about having a friend (girl friend?), what do you think of that? You might not start but what type of friend you wish to have for future?

R: a woman like me (to mean HIV positive) or who is healthy and know about me and accept my status (to mean HIV negative)

I: ok you think that, but what is your first choice? That means whom you think first? Not from face but from health side?

R: whether she is negative or positive it has no problem for me

I: why not has a problem?

R: since I am already positive, whether she has or not, it doesn’t have a change for me

I: but doesn’t a relation with whom she has and not is different?

R: it is different I think it is with doctors’ permission to make a relation with whom she has

I: what if she has no?

R: it also be with doctors’ advice that having child be possible

I: aha aha you thought that, so for the time you have no a relation (girlfriend)?

R: yeaha I don’t have

I: at what age do you think you will have a friend?

R: when I have job and around 28 years old

I: ok what is your future; what do you want to be?

R: I want to be a driver like my father, I end my grade 10 education and I will have a driving license

I: ok what other things you wish to be done for you?

R: I will be happy if the six month’s HIV vaccine comes. If it comes it will be good for job and other things. So I wish if it comes

I: ok what else? Earlier you said me there is a youth club; have you quitted it?

R: yeaha, it is not good

I: why?

R: all have different behavior which is displeasing, so I don’t like that

I: Don’t you think it is beneficiary?

R: it has a benefit, they teach as many things but due to that I stopped it

I: ok I finished my questions thank you very much I may revisit you if there is missed thing

R: ok
