# Supplementary material for: “I want to perform and succeed more than those who are HIV-seronegative” Lived experiences of youth who acquired HIV perinetally and attend Zewditu Memorial Hospital ART clinic, Addis Ababa, Ethiopia
Source: PLoS One. 2021 May 27;16(5):e0251848. doi: 10.1371/journal.pone.0251848 (PMC8158987; doi:10.1371/journal.pone.0251848)
Supplement: S1 Data — (ZIP) [file pone.0251848.s003.zip › S1_Data/5F22-05 word.docx]

**Study Title: Lived Experiences of Perinatally HIV Infected Youths**

**Unique ID**: F22-05

**Interview category**: In depth interview

**Interviewer name**: Nahom

**Interview date**:

**Interview duration**: 39’:46’’

**Place:** Addis Ababa

**Transcriber name**:Nahom

Section1:- Socio demographic characteristics

I: ok your age?

R: age 22

I: your education level?

R: advanced diploma

I: ok occupation

R: I am working, I am a teacher in other department, I have graduated in hotel and tourism

I: marriage?

R: I haven’t married (laugh)

Sex: female

Religion: Orthodox

I: ok with whom are you living?

R: I live with my father and stepmother, I have also brother

I: what about your mother?

R: she has died

I: ok, how long is since your mother has passed away?

R: when I was 5 years old

**Section 2:- health status**

I: how is your health as general, what do you feel beyond issue of HIV?

R: I don’t feel anything, I am very happy

I: when did you know as you have HIV?

R: as I have HIV…I mean they didn’t tell me at home, it is here by myself, I mean from what I hear and I see, I used to suspect, they didn’t tell me at home but when I came here and asked, one woman told me

I: ok I mean you had been taking drug before you knew that you had HIV?

R: Yeaha I have been taking and I used to ask my father repeatedly why I took the drug, but when I asked him he gave me something else, since I had had a repeated cough and the like, so he answered me as it is for cough and something else. So since I used to think such things, one time I came here (hospital) alone and I got one woman alone, at that time I was very kid and she asked me with whom I came, I said ‘I came alone’, then she said “so do you know this(the drug)” I said ‘no I don’t know but I think it is cough medication’, she said “no it is not” and she told me for what it is.

I: ok what did you feel at that time? What was your reaction?

R: since I used to suspect, I almost knew it by self; I didn’t feel anything. I also hear from media in which they say time should be strict and something related to taking drug, since I used to hear such things, I didn’t feel anything

I: it was when you knew well about HIV?

R: around early of 10 years of age

I: how you understood and what have you thought when you well know about HIV?

R: HIV is nothing

I: is it from education perspective or is your internal feeling? The education says that but I need your real reaction by that time. What did you say when you heard that you have HIV? What have you said to your father?

R: I didn’t say anything to my father, I wrote on my diary and it was after that he had read my diary and he understood as I had known it. Otherwise I didn’t tell and talk him; because before that I repeatedly asked him; “this disease is this this so answer me” and many time said me it is not and he denied many times, he was not willing to respond for me.

I: ok so what he said when he understood as you knew it?

R: he said nothing

***Section 3:- supportive conditions***

I: what are the things you think are supportive for you?

R:…….(silence, nodding head as no)

I: nothing? Let for example from family side?

R: from family…I mean we are not many, since father has another wife they see me as additional, father focuses on them (new family), it is not on me. So we are not that much with family, but we talk with friends, I have one close female friend (she is also HIV positive), so we talk everything.

I: who knows about you?

R: about me only those who are here (hospital)

I: what about family?

R: family….my father and the stepmother

I: others?

R: no other person knows

I: what about your brother?

R: my brother is 12 years old, he sees when I take the drug but don’t know why it is for

I: ok what else? From your working area and neighbours, who knows?

R: no one knows, but I had had a boyfriend and since I didn’t want to hurt him, I have told him, we agreed and he knows.

I: ok is that?

R: yeaha

I: what other thing you say “if this is done, if it is done in this way, it would be good for me, it would support me?”, is there anything you think it should be done and supportive for you?

R: I mean awareness of most people is somewhat lower, actually it is better than formerly but still they lack much, they view HIV as other thing bad and we, we children most of us didn’t bring it by ourselves, we inherited from parents; they don’t think that, if you see in our family; my father’s wife (stepmother) doesn’t have good view, she is not happy of me and she is not that much happy by what I use. So I will be happy if such things are corrected.

**Section 4:-concerning issues**

I: ok what are the things you are concerned of related to your health?

R: I will be happy if the medication is gotten

I: what type of medication?

R: that of HIV (laugh)

I: what about the drug you are currently taking?

R: no, no the medication?(laugh)

I: so what type of medication?

R: which can totally cure, we heard that; there is an injectable

I: who told you?

R: …….(laugh)

I: I mean it is new for me

R: no it is long time since we heard of it

I: have you heard from health professionals?

R: yeaha, here(hospital) we used to learn in youth club( a program where HIV positive youths get together), now they excluded us (above 19 years old are obliged to leave the program), so in that program they used to say that and gave us hope as it comes, but still it didn’t come

I: what other things you think are concerning issues; like from living and working environments?

R: since no one knows about me, there is nothing which concerns me

I: when you see messages about HIV from medias and posters, what feelings would they create on you?

R: they (HIV messages) fluster me (yanadidegnal)

I: why?

R: I don’t know

I: Is the way they present and describe HIV is not right?

R: it is not being right or not, I don’t know but only when I see the HIV messages, I become angree, honestly speaking I don’t feel good thing

I: so are you saying it should be disappeared or corrected?

R: I don’t say it should be corrected, I don’t know but sometimes I don’t like to see such messages (HIV messages); if I came across with posters of HIV I may see the first word and I don’t finish it. At all when such messages (HIV messages) are presented on medias, I am not happy; especially when I sit at home with family I loss my internal peace

**Section 5: challenging things**

I: are there things you think as challengers for you? You already told me on related with family

R: family…yeaha, if you are strong you will cope it, it is what you can pass

I: ok what else? Like from taking your drug side? Can you take your drug freely? Is there a challenge related with that?

R: from taking the drug side; I mean it may be convenient or not but I take one drug covering with paper everywhere I go. When I say this, I may take it or not; if it is convenient I may take it otherwise not

I: convenient means? Is it if people are not around you?

R: yeaha, like that; because no one knows about me. I have told you who knows; only 4 or 5 people, even those are family members, so if it is convenient I take otherwise I take it going to the rest room

I: when do you think children who had gotten HIV from parents should be told their status?

R: when they are 10 years

I: 10 years? You knew around that age?

R: yeaha, 10 or 11

I: so do you think you have benefitted or hurt due to knowing in that age?

R: not telling me hurt me

I: was it better if they told you before that?

R: yeaha, because I used to suspect, you know when you become ill there is something that you think internally, additionally in that time many things used to be said about HIV; as it is killer, people infected of it are emaciated, so since I heard that and also read, since I see that, it would be better for me if father told me. Even not telling me hurt me much. Even I became angree and discontinued my drug. If he told me I wouldn’t be anything, because I took it for my selves, it is not for him. If he told me, I thought I would be benefited but not telling me hurt me much

I: was there a time when you have discontinued your drug?

R: yeaha

I: for how long?

R: it is not for long time but for 15 days, for a week something, I discontinued my drug

I: why? Due to what?

R: when there is conflict at home

I: so do you say ‘I leave it something?’

R: they (family) said “we don’t care it is your business”(laugh), telling you the truth; they don’t care they say “if you need take it (the drug), otherwise it is up to you, we don’t care it is your business”, so one time because of a conflict I was not home, I was with my female friend and since I left the home without the drug I discontinued it for 15 days. Again my friend doesn’t know about this and I knew that she has a very bad thought about HIV, I couldn’t disclose to her, after that I started.

I: does your clinic appointment clash with job?

R: no it doesn’t since I come on Saturday, it doesn’t clash

I: no problem? Can you take any time convenient for you?

R: yeaha I can take my drug any time from the clinic

***Section 6: sexual behaviour and relation***

I: ok you have touched it above. You know when age increases there is also related changes? What do you know about reproductive health?

R: here (in the hospital) they advise us about drug, life, what type of friend should we have and the like

I: what is your thought about relationship? What did you have before and what do you think for future?

R: what you mean by relationship

I: I mean relation with male

R: I told you earlier, we stayed long (with her boyfriend); around 3 years and more, I told him, he believed in and accepted. But now we are not together

I: you mean have you discontinued or other thing?

R: I don’t know why that happened, his phone is closed and it is around 3 months

I: up to what level have you gone?

R: ……..(laugh), we went up to end, I gave him everything, I don’t know anything without him, I gave him everything. But he used tom be tested every 3 months but what confused me is that he is free of HIV (negative)

I: but he believed in and you didn’t use condom?

R: yeaha he believed in and we did without condom

I: why he didn’t use condom

R: he refused, he don’t like it

I: ok if your friend returns you wish to continue but what if he doesn’t return? What do you think for future?

R: I don’t think anything; to tell you the truth I love him very much, I am happy if he returns but if not I am female, I can’t live alone; seeing who may benefit me I will marry.

I: ok whom you marry? is one who has or not (HIV positive or negative)

R: I prefer one who has (HIV positive), I will be happier if that is. But again if one who believes in, understands, accepts and willing comes, that is ok.

I: you have been together for more than three years and practicing sexual intercourse without condom?

R: yeaha we did without condom

I: why don’t he uses condom

R: he doesn’t like it

I: what do friends like you (who are HIV positive) think of about sexual relation?

R: you fear starting relation. You know when males come they usually ask you for sex so you fear and go, I have faced such things many times, it is hard to disclose yourself rather you escape.

I: what do you talk about sexual relation in your youth club? How do youths pass through it?

R: we don’t usually talk with all, you know since it is young age they went to different relationship. Some don’t see sex as big issue, they do it, there are youths who practiced unsafe sex and later said ‘I have done and it regrets me’

I: don’t they take care of?

R: yeaha

I: why?

R: you know most of them are youths and some may be addicted, due to such things they might do it and they told us after it is passed. They told us as they did a mistake and they regret. Once they love some females can’t quit, so there are who engage by hiding themselves

I: do you think you have benefitted because of disclosing yourself for your boyfriend?

R: yeaha; first I felt rest, I mean I stayed long months without telling him before starting any sexual contact but after he asked me for sexual intercourse, I felt a lot and I thought if we engaged to detail he might be infected; so if I didn’t disclose for him at that time it would regret me. Whatever it is, since he entered knowingly and believing in it; now it is a rest for me.

I: ok what other things you would like to add on what I asked you?

R: to teach the community about disease

I: what do you think is your role?

R: if one discloses himself what role will he has? You know when you argue about the issue; there are people who say “are you also positive?”, so you fear such things.

I: is there anything you have ever faced?

R: my female friend where I sat for 15 days, you know she has bad attitude to the disease; if you ask her what she may do if she has HIV, she says “I kill myself” for your surprize she uses all my materials including clothes but she doesn’t know my status, if she knows that; I know she will not stay with me.

I: what do you wish to be in the future?

R: after this? what is left…….(laugh)

I: ok have you gotten what you need?

R: no I wished to be a doctor but…..

I: so why not now

R: after this….(laugh)

I: why not? any ways I have finished my questions thank you!

R: ok thank you!
