# Supplementary material for: “I want to perform and succeed more than those who are HIV-seronegative” Lived experiences of youth who acquired HIV perinetally and attend Zewditu Memorial Hospital ART clinic, Addis Ababa, Ethiopia
Source: PLoS One. 2021 May 27;16(5):e0251848. doi: 10.1371/journal.pone.0251848 (PMC8158987; doi:10.1371/journal.pone.0251848)
Supplement: S1 Data — (ZIP) [file pone.0251848.s003.zip › S1_Data/6F19-06 word.docx]

**Study Title: Lived Experiences of Perinatally HIV Infected Youths**

**Unique ID**: F19-06

**Interview category**: In depth interview

**Interviewer name**: Nahom

**Interview date**:

**Interview duration**: 42’:03’’

**Place:** Addis Ababa

**Transcriber name**:Nahom

Section1:- Socio demographic characteristics

I: how old are you?

R: 19

I: 19? Ok, your education level

R: I am college student

I: degree?

R: yeaha

I: year?

R: first year

Sex: female

Religion: Orthodox

Marital status: single

Occupation: student

I: ok with whom are you living?

R: with my aunt

I: is your father alive?

R: yeaha but we are not together

I: when did your mother passed away?

R: when I was kid, I mean when I was 1 year old

**Section 2:- health status**

I: what do you feel about your health as general? I mean your health beyond HIV?

R: glory to God still I am fine, before I started to take the drug I had had experienced disease of TB and others. But after I started the drug I am peace except headache and abdominal cramp as anyone else.

I: ok how long is since you have known that you have HIV?

R? It was told me soon

I: how long is?

R: about 10 years some thing

I: so was it when you were 9 years old? How was the incident?

R: as I told you I used to be sick, and I came here (hospital) incidentally and made to follow this thing; I mean the treatment. Then they have counselling and I entered there, education is given there, still also found, and I knew it after that. It was my aunt who told me. Sometimes silently taking drug creates a question on you. Since I was asking she told me. So since she had told me as anyone would be caught and since I also watch from television and knew, again since I used to participate in different clubs it didn’t worry me.

I: so since when have you known your status well?

R: when I was 9, 10years some thing

I: that means was it the first time you have known?

R: yeaha, I was kid

I: I see, you have started the drug before you knew HIV in detail, and then you asked why you are taking the drug?

R: I didn’t stay much; I didn’t stay much after I started the drug

I: have you asked or they themselves told you?

R: no, my aunt herself told me

I: at home herself?

R: yeaha at home

I: what was your feeling at that time? What was your reaction?

R: at one time you may….

I: I mean at that time when they told you?

R: since I was kid I didn’t feel anything, you know you feel when you grow, but for me since my aunt supported me I didn’t feel anything.

I: what you felt when you grew?

R: sometimes you can’t be as others

I: you mean?

R: for example if you see relationship unless with a person like you (to mean HIV positive), you fear to start with one who is negative. It is not starting the relationship that fears you but after staying long time together and when you told your status, that person may not accept you. Actually now it is said that most people could understand but it is not, most people’s thinking is weak.

I: is there anything you have ever faced?

R: yeaha there is; I mean I told such thing, when you start relationship as we are teenagers, so when you start this thing, mostly I don’t start but at one time I engaged quietly, then when you stay long time and the relationship became strong you fear, at the end I told him but then the feeling was very bad and due to that since his thought was yet null you fear to tell, you fear to form a relationship.

I: but before you told him, haven’t you seen his feeling? How was it?

R: I had asked him, I mean at different times we used to talk like when we see from poster; we talk but sometimes you don’t live what you talk.

I: was his thinking positive when you said him ‘if I have something’?

R: yeaha, he used to say “what is the problem, as anyone something” but we human being since we don’t live what we speak, when you told as you are HIV positive the response is very bad.

I: ok when do you think is better to tell their status for children infected from parents? At what age is better?

R: you mean?

I: as they have, for example you knew it when you was kid, was it helpful or not for you?

R: it benefited me, it benefited me very much, because if they didn’t tell me and I kept on taking the drug quietly, I might not know the way it is prevented and transmitted. You see? You keep quietly, I may hurt and others too. But telling me in that time helped me to know how it might be transmitted. Since I knew that I also knew not to hurt myself, just you save yourself from many things. So mostly if it is told when most children became aware and able to understand; I think telling has a benefit. Even family is peace, here more than the sick person it is the care giver who worries. If they told; I think it is a benefit for children and also for all.

**Section 3:- Supportive conditions**

I: let what are the positive or supportive things you have around you?

R: is from people’s thought side?

I: for example from family, school, friends and the like perspective; what are the things you take as supportive for you?

R: it is good, as I told you I am living with my aunt. She has also HIV; we are living supporting each other. We used to come here together but now when I grew, she perceived as it may discomfort me and left me to come alone, when she goes to country side I take her drug home and we support each other very much. I have families, I have brothers; all the families know my status, they all support me. Since I am young they support me in many things, they give me all cares

I: what else what is the thing that gives you rest and pleasure?

R: I don’t know but mostly I go to church (bebizat betekrstian new yemihedew)

I: what about from school side?

R: school? But here at hospital, as I told you we have counselling group, I left it recently, you will leave if you are above 18 years of age

I: why those above 18 are obliged to leave?

R: it is so as those little children come up, you know if all stay; we are going to be many and you are going to learn repeatedly what you have already known before, I mean you take what you have already taken.

I: ok but what about your relation beyond the education?

R: we want to meet; I mean even if it is not here at hospital still we those who left the club also meet privately. Because we stayed here long time and when you stay long time you will be as sister and brother so we thought to be together, so we meet each other. Taking tea and coffee; we talk about our life, what we have faced and about our future.

**Section 4:- concerning issues**

I: ok what are the things you think as they are concerning for you?

R: what concerns me is my life, I mean my future life; I am human being and when you grow and reach at some level you will form family; so at that time as I told you there is a man who has good thought and bad thought too. If God helps you, you may get one who has good thought, so this thing concerns me very much. I mean sometimes I feel as I may remain alone; I mean without forming family something. You know the community is yet, doesn’t know anything. They don’t believe that we are able to do as any one. Currently there are many diseases which don’t give seconds for people; so many things should be done about this disease. People should be awared, so my future life concerns me.

I: beyond that now there are messages about HIV on medias, schools and clubs; so how do you see such messages? How do you understand them? What feeling do they create on you?

R: some are educating; I also did in HIV clubs at school, sometimes when you teach people you feel rest, but now it is forgotten on medias, since the kidney disease came, most even on face book the issue is it. It was formerly when education was being given but now it is forgotten; I can say 100% it is stopped, it is not being done at schools, they forgot it, so they forgot the disease; the disease is forgotten. But at this time the disease is being spread more, so I think much should be done.

I: how do you see the way HIV is being described? Do you think they are right or not?

R: I think they are right, I mean we usually hear about prevention and transmission methods like mother to child; they are nice but should be done broadly. I mean here it is not because you eat and drink, there is what is called future; now I fear everything in the future will be done by testing, honestly speaking you fear to apply for job, it is because I faced such things, there are people in my residence area; they fear very much.

I: on what type of job is?

R: for example see any type of job; like to go foreign country, again on others you will be asked for medical certificate, even you will do that for driving license, so when you do medical you fear. At one time blood and urine will be taken so at that time you fear.

I: but HIV is not asked

R: yeaha it is not asked, I mean that of future; it may be asked. Honestly I do have fear, so for us HIV positive people everything should be without threat, even to form life (marriage) it should be without threat. So that will be happen when medias and people work but our community is yet, very yet as formerly

***Section 5: challenges***

I: what are things you think challenging for you? Beyond the community’s awareness you told me what you say are challenging?

R: what I say challenging; my residence area is far from the clinic. I come here not because of lack of health centre with the service there; first here in the hospital I do have long time follow up experience and they had also told me as I can make my follow up at nearby health centre, but you fear. For example I take for my aunt from the nearby health centre, when I go there what is displeasing is the clinic is separated from others, they separated HIV positives’ clinic. Even this building (at the hospital) is identified and known as it is ART class. Formerly we used to take silently entering in but now it is separated. So when I sit to take for my aunt I fear, it is not for me but what she will feel if she goes there, so sometimes not to disclose her I collect her drug. (ye ersua sim kemitefa eyalku ene medhanitun ewesdlatalehu). So when people see you they thought as you take the drug, even a person who sit in your side will talk about you and gossip with others just making himself as he has no HIV; he will say “I see her when she take drug” and something like that. So I will be happy if the clinic is not separated.

I: you mean if it is as any other units together?

R: yeaha I will be happy if not separated because when it is separated people will have suspicion, when you enter they thought as you are entering to ART, they don’t say as you enter to emergency class. So I will be happy if such things get correction

I: what else, for example can you take your drugs in your time even where there are people freely?

R: it didn’t face me; I take my drug two times a day but I take it in my private class. I put it locked, if people come I will take it going to my aunt class.

I: do you have a fear if people see you

R: yeaha it does

I: with whom do you talk about your health?

R: mostly I talk with my friend whom I got here (she is HIV positive), and another is my aunt used to ask me when I was kid but now it is not that much

I: what about your father?

R: I don’t meet my father that much

I: Doesn’t your father know your status?

R: he knew but he doesn’t say me anything

***Section 7:- sexual behaviour and relation***

I: you know when age increases there are changes which come together, so would you tell me anything you know about reproductive health?

R: we learned at school clubs, we have also taught and it is also given here in the hospital; due to that I know about reproductive health

I: in your clinic appointment do you only take drug? Don’t you learn about reproductive health?

R: yeaha we take only drug, and weight may be measured

I: what do you think about sexual relation? What do you think for future and is there anything you have started?

R: up to now it is as I told you; since then I am not that much. For the future it is God who knows. We are human, we try. You don’t sit saying I don’t love. Saving yourself; it is not being in a hurry.(rasihn save adrgeh, le negeroch alemechekol new) I mean it is a pleasure when everything will come on its time. Even if you love I should not hurt that person and he shouldn’t also. I will be happy if we are similar (both HIV positive)( bihon bihon and aynet binhon des yilegnal). But if not just if he has awareness and accepts me that is not bad (ginzabe kalewna silene yemikebel kehone esu kale aykefam)

I: ok you think that?

R: yeaha but you are human and try; it is when you try you get something. It doesn’t come if you sit silently. Since love is incidental I can’t sit saying I can’t love. But if the incident happens, I am sure I will tell the truth; there is nothing I hide, because it is not in which I will ashamed of, it is not happened and came because of my choice. So since I can be as any other person, I will tell him, if he doesn’t accept that is not, and but if he accepts that is good.(kaltekebele altekebelem new, ketekebele demo eseyew)

I: do you think the youth club helps for this?

R: yeaha so much, especially if you are the same age group there is a chance of being together. It is divided in to two; for elders and little children. As I told you elders don’t detach each other even if we excluded from the club

I: how many are you who meet personally out of the youth club?

R: we are many both female and male; when you meet, when we get together; since we share ideas, you may start relationship too. It is as chance, so it is nice. It is very very nice for us; from school side and getting friend side it is very nice. For your surprise children who live in the same residence area knew each other here in the hospital. You know I shocked when I see them at first time but later we became best friends. We talk even going to at home; one day in one and the other day in once home.

I: what do you think is your role in preventing HIV?

R: I will be happy if I can change the community; though not the whole but one who is around me; I mean you start from home then you will move to neighbour. So I will be happy if I could do that, if not I need to continue saving myself and person around me who hasn’t has HIV. Because after this, since it (the HIV) doesn’t leave me even if I say it; I feel to continue doing that way.

I: haven’t you ever heard who have cured?

R: I heard by that of spiritual water (tsebel something) (betsbel mnamn sibal esemalehu). You don’t know that is God’s work. The major thing is to believe, if you believe it will be. It is because we are weak, but if we believe and have pure heart it can disappear, it is God’s work.

I: what do you wish to be in the future, where do you want to reach in your education?

R: I like business; even now I started something personally. I am working and learning. In my education also; as any other person I want to reach at big level. Even I want to be above who has no HIV. If you do that you will get the chance to educate others.

I: you have a nice moral; there is no reason you may not reach that

R: as per God’s will

I: what other things you wish if they are done is good for you and children like this?

R: you know this youth club counselling found only in the middle city, it should be spread, it should be given at health centre level because people at far don’t come here; mostly they are treated at nearby health centre. They should know each other, they should support each other. So it is when you do that you keep yourself and develop a relationship, otherwise you go in fear. But if there is counselling club you meet a person whom you might fear at distance and be sister and brother. So such things should be done for children who are HIV positive. The other thing is we have to aware people, the community should learn much, and awareness should be given very much.

I: is there any way you suggest? Formerly people have been disclosing themselves and teaching; saying “let it ends on us”, so do you think it should be done now too?

R: I don’t think we dare to do that now, you know there are people who tried but failed and again there are people who fear to be seen in the community by this way. As I told you there are people who fear how to continue their life so medias should add new things beyond routes of transmission and work more. You know there are many things we need, so better if they present all these and education be given well.

I: thank you very much I have finished my questions!

R: ok me too thank you!
