# Supplementary material for: “I want to perform and succeed more than those who are HIV-seronegative” Lived experiences of youth who acquired HIV perinetally and attend Zewditu Memorial Hospital ART clinic, Addis Ababa, Ethiopia
Source: PLoS One. 2021 May 27;16(5):e0251848. doi: 10.1371/journal.pone.0251848 (PMC8158987; doi:10.1371/journal.pone.0251848)
Supplement: S1 Data — (ZIP) [file pone.0251848.s003.zip › S1_Data/9F21-09 word.docx]

**Study Title: Lived Experiences of Perinatally HIV Infected Youths**

**Unique ID**: F21-09

**Interview category**: In depth interview

**Interviewer name**: Nahom

**Interview date**:

**Interview duration**: 51’:24’’

**Place:** Addis Ababa

**Transcriber name**:Nahom

Section1:- Socio demographic characteristics

I: How old are you?

R: I am 21 years old

I: ok your education level

R: I am grade 11

I: are you learning grade 11 now?

R: yeaha

Sex: female

Religion: Orthodox

Marital status: single

Occupation: student

I: with whom do you live?

R: with my sister

I: ok with your sister, your mother and father?

R: my mother died when I was kid, when I was around 4 years of age and it is around 9 years since my father has passed away

I: are you now living with your elder sister? Does she have also?

R: no she is healthy; it is only me from the family who has HIV?

I: ok only you; do you know as your parents have died from HIV?

R: yeaha I know

***Section 2:- health status***

I: ok please tell me what you feel of your health as general?

R: you mean?

I: how is your health as general beyond the HIV condition, have you ever faced any big health problem?

R: glory to God still I am well, sometimes there are stress and mild diseases like cough, malaise, shortness of breath and skin disease, otherwise I am fine

I: ok have you ever had big health problem for which you were admitted at hospital level?

R: no

I: when have you known as you have HIV?

R: when I was 14 years of age

I: when did you start to take the drug?

R: when I was grade 5

I: now you are grade 11,that means when you were around 14 years of age? That means have you started soon when you knew your status?

R: it is not, it had 1 year gap,

I: how was the incident? How you knew your status?

R: my father used to take drug and when he died I became sick very much repeatedly, so all the family member decided to be tested and I was also tested.

I: ok have you tested here in the hospital?

R: yeaha

I: what have you felt by that time?

R: mine is somewhat special, I used to read and wanted to know everything, though I didn’t think if I have it, but I used to think of what care should I do. But when you know, when you hear you will be shocked (sitsema gn ye hone tidengtaleh)

I: what was your reaction?

R: I remember I cried, but after that I remembered from what I read and I calmed myself, I convinced myself as I can live. The nurses also advised me and my family supported me a lot.

I: who knows about your status?

R: all family member know

I: why you started the treatment after one year passed from knowing your status?

R: the reason why I didn’t start at a time was; amidst due to father’s death our life was in danger; up to missing residence, for that reason we moved to countryside, and you know at such place there is no well-organized services so I couldn’t start soon after I knew my status. So after a year I went to a nearby city and started to take the drug.

**Section 3:- supportive things**

I: what are the things you take as supportive for you in your environment?

R: I don’t understand

I: for example let take from family side; what are good things for you from them?

R: family…they are not happy of my status, I mean they wish if I was not. But they support me in everything. I also don’t think in that way

I: what else? Like from clinic side what positive things have you seen?

R: there was a so called peer counselling; so since we the same group get together we talk everything and share our feelings freely, but now it is not there; I wish if I join it. The other thing is doctors also have good approach but sometimes they don’t give you time, they don’t talk you politely and the like. I think they have many clients, even they transferred me in after much tribulation, refusing me saying as they have many clients.

I: what else like from school and neighbours’ side?

R: since no one knows about me I haven’t seen any supportive things

I: how do you see the way HIV is described in medias and the like?

R: nowadays I think it is forgotten, you know people don’t give attention, relative to the time when I started my treatment now the attention being given is low; they see it as cough. They consider it as something simple; I am not saying it is frightening but it should get attention, otherwise it is difficult. It is said’yenakut mn mnamn yemibal ale aydel?...(laugh) which to mean something which is under estimated may harm.

**Section 4:- concerning issues**

I: what are the things you take as concerning issues for you?

R: by this time what concerns me is my education, I wish to join campus but I worry of what may I do if I fail? I can’t say I will go abroad or other things as others, or I can’t also do labour work, so when I thought that it concerns me; so I focus on my education

I: what other things you wish to be done for you?

R: I wish if there is a program where I meet people like me (HIV positive youth), at least one day where you breath, where you speak your internal thought and go. It is hard to talk at home where people around you are negative; you fear and even your thoughts don’t get each other, but if you are the same, you can talk everything you need, you can say “we”. So additionally it is good if the education be given at each level. In schools and the like; as I said you, it is forgotten.

**Section 5:- challenging things**

I: ok what are the things you say as challenging for you? You have told me early that of family issues, what else? What challenges have you ever faced?

R: the challenge was sometimes when you go at hospital and sit at waiting room people around you judge you; you know they consider you as bar lady and the like, even they ask you for such things there. One of my experiences was; one time when I was at waiting room, a woman on my side asked me if I got it due to sexual contact; I stunned and asked her why she said that. It was because I was youngest than all who were around there. And I said her no it is from parents. So what I think if it be is; our clinic should be separated from adults.

I: doesn’t it is separated here?

R: it is only the class which is separated otherwise we sit together in the same side at waiting area. The other challenge is; our card is located at one place at hospital level. So I wish if our card is put separately and located near of our clinic, because it is challenging. You know you may loss your card repeatedly and due to that you may not get the service you need on time

I: does your clinic appointment clash with your class? Have you ever missed class due to clinic appointment?

R: sometimes it happens, for example today I had had an exam and I came here after taking it

I: what do you do when your class and clinic appointment clashes?

R: I may come before or after my schedule, I may come on Saturday

I: but was there a time when you missed a class? What do you say to them?

R: I may take sick leave and simply I say them I was sick.

I: how do students around you describe HIV?

R: they see it as a very simple thing, you know all take it as a very minor thing; even I ask my self-saying is only me who think of it?

I: when do you think is the right time to tell for children who acquired HIV from parents?

R: I think though they don’t be told they should learn it since child hood, if they get the information what to do if they have and at the same time what to do if they don’t have. And I think it is good if they know after their 14 years of age.

I: ok how many times in a day do you take your drug?

R: one time

I: ok can you take freely when people are around you?

R: yeaha, you fear but you can’t do anything

**Section 6:- sexual behaviour and relation**

I: the other thing is you know when age increases there are related changes, so what do you know about reproductive health?

R: I think I do have enough knowledge, you know I have participated in different trainings and even from school I know well.

I: ok so what is your thought about friendship? What is your experience and what do you think for future?

R: I had had a friend (boyfriend), but it failed

I: how long have stayed together?

R: it was short period; once there was a conflict in the family and due to that I was not at home

I: ok have you been with your boyfriend?

R: yeaha it was not that much which we talk for, it was not good. We were at fire age and we did it for satisfying our sensation

I: did he know about your status?

R: yeaha I have told him before we did anything, and he was also positive, his mother and sisters too

I: had he acquired from parents?

R: I think so but he was young and ….(silence)

I: I understand you it is not to disclose your secret but it is because what you have really experienced is supportive for us, so feel free and tell me what happened? How old was you by that time?

R: 17

I: how long have you stayed together?

R: we stayed for three months in one home like husband and wife

I: was the sexual intercourse without condom?

R: yeaha we did it (sexual intercourse) without condom……..(laugh)

I: I understand you it is as I said you there is nothing that embarrasses you, so what was the know-how you had had, that means though the two are positive what is recommended about sexual intercourse? I need not the education being given but the thought you had had at that time

R: initially we didn’t make any care; he said “since we are the same there is no need to do any care”. There was nothing I knew

I: ok have you stayed 3 month in that way?

R: yeaha

I: but what is advised about sexual intercourse even for those who are HIV positive?

R: what is advised is even if both are HIV positive they should use condom because the virus in both is not similar and it could be transmitted from one to the other. So we should made a care but…..(silence)

I: ok what is your thought about friendship (boyfriend)?

R: before that I have a message for parents. You know parents should treat their child during their fire age. They should listen their feeling and approach them kindly. They should not be serious and ignore them. If they ignore them their destiny will not be good. So once things are deteriorated it is difficult to correct. Usually our parents try to work after many things have passed. If you take my parents; they were very serious and didn’t listen me and that was why my life went in that way.

I: ok now do you have a boyfriend?

R: there is a guy whom I love. I love him very much and he knows everything about me. I told him as I am HIV positive but he is free. Actually we don’t know each other in person; it is via face book and we are a friend thinking to be together.

I: did he accept that?

R: he accepted and he told me as it has no problem. But since we didn’t meet in person you can’t be sure of

I: beyond that what is your thought about sexual relation for future? What would you do if this fails?

R: I think a guy like me; but it is difficult to get that. You know love is incidental, so it is hard to get a person like you. Sometimes I moved around here (the hospital) saying my fate is here. But again I say it is not something I could get by finding. If I get someone who is the same with me (HIV positive) it is okay otherwise I prefer to be alone.

I: do you disclose yourself for one who is negative

R: no I don’t need HIV negative, I don’t want to hurt other

I: but your current boyfriend is HIV negative

R: yeaha it is because I love him first, it is difficult but I don’t want to hurt him too

I: so what would you do?

R: I will quit it. I don’t want to see him being like me. I will leave him creating some reason. I love him very much but I should sacrifice for him by leaving him. I never hurt him.

I: what additional things you want to say?

R: I have no additional but as I told you I will be happy if our clinic is adjusted for adults and youths separately. The other thing is I wish if the youth club is there for us because it is very much helpful. Since we are the same group it is easy to talk with them freely. They understand you and again it is helpful to get boyfriend and girlfriend.

I: what do you want to be in the future?

R: I want to be psychologist; I want to work on prisoners and students. I want to work on forgotten people. You know when you close and talk them; you get what you didn’t expect. They want to share their ideas but they don’t get who hear them. So I will be happy if I reach them and give them what can I do.

I: what do you think is your role in HIV prevention?

R: firstly I should take responsibility for myself and second I should take care of all people around me. I should not allow others to be hurt.

I: ok thank you very much wish you all bests

R: ok thank you
