# Supplementary material for: “I want to perform and succeed more than those who are HIV-seronegative” Lived experiences of youth who acquired HIV perinetally and attend Zewditu Memorial Hospital ART clinic, Addis Ababa, Ethiopia
Source: PLoS One. 2021 May 27;16(5):e0251848. doi: 10.1371/journal.pone.0251848 (PMC8158987; doi:10.1371/journal.pone.0251848)
Supplement: S1 File — (DOCX) [file pone.0251848.s004.docx]

## Annex-I: Study participants’ (youths’) information sheet

Greetings! I am Nahom Solomon a masters of public health student from Addis Ababa University, currently am doing a health study about HIV/AIDS and related health issues, specifically among youths who acquired HIV from their parents. The study concerns about supportive conditions, concerns about, related challenges, and sexual behavior and relation of youths, generally which is about lived experiences of those youths who are on HIV treatment follow up.

**Aim of the study**:- Its aim is learning the lived experience of youth who acquired HIV perinatally, so that the finding will be helpful in designing better health care services to improve the health status and control HIV transmission.

**Process of the study:-** The study includes those 15 up to 24 years of age. To be part of this study, you are selected randomly from all other people like you. No laboratory or other measurements are needed; you are only expected to freely discuss with the interviewer. The conversation may take about an hour and for missed information and further clarification you may be re visited as needed in another day based on your willingness.

**Rights of the participants:-** Your participation is fully based on your willingness. As all the conversation is up to your willingness, you are fully entitled to ask, interrupt, skip questions and withdraw from the study any time you like.

**Confidentiality of the study:-** In any means the information you give will not be used for other purpose beyond this study and always be kept in confidential. During the interview, if you are willing, I will use an audio recorder, which means that what we talk about during the interview will be recorded. This is so that I can remember what we talked about. There is no need to mention your name or other identification. The audio tape will be kept locked in a cabinet in my house and only the researcher will be allowed to listen to the audio tape. It will thereafter be destroyed.

**Benefit of the study:-** Being participant of this study by itself doesn’t have a direct benefit for you. However this doesn’t mean it has no benefit at all. As tried to mention in the beginning your information is helpful for improving health care services. At the end of the interview session we will have tea and snack together.

**Risk of the study:-** Your participation has no risks, in all means you are free of any harm and for that the researcher is responsible and accountable.

So considering the above issues I kindly request to put your response in the next page of consent form. If you have any questions you can contact me through the given address.

Thank you!

Nahom Solomon

Cell phone:- +251941246518

Email:- [nahomsolomon83@gmail.com](mailto:nahomsolomon83@gmail.com)

Addis Ababa University School of Public Health

**Study participants’ (youths’) Informed consent form**

I read/listened the above information and I understood that it is a study that doesn’t harm me, is based on only my willingness and promise confidentiality of my responses and no harm and special benefits to me. Accordingly based on my understanding, regarding my participation on the study, without any pressure I reached on the following decision.

1. I fully agree to participate
2. Do not agree

**Interviewer**

I assure that I informed and took the consent

Name: ________________

Signature: _______________

Date: ______________­­­­­­­­­­____

## Annex-II; Parents’/Guardians’ Study Information Sheet

Greetings! I am Nahom Solomon a masters of public health student from Addis Ababa University, currently am doing a health study about HIV/AIDS and related health issues, specifically among youths who acquired HIV from their parents. The study concerns about supportive conditions, concerns about, related challenges, and sexual behavior and relation of youths, generally which is about lived experiences of those youths who are on HIV treatment follow up.

**Aim of the study**:- Its aim is learning the lived experience of youth who acquired HIV perinatally, so that the finding will be helpful in designing better health care services to improve the health status and control HIV transmission.

**Process of the study:-** The study involves those 15 up to 24 years of age. To be part of this study, your child is selected randomly from all other similar people. No laboratory or other measurements are needed; it is only expected to freely discuss with the interviewer. The conversation may take about an hour and for missed information and further clarification you and he/she may be re visited as needed in another day based on your and his/her willingness.

**Rights of the participants:-** Your child’s participation is fully based on your and his/her willingness. Actually although you are willing for your child to participate he/she can refuse to participate irrespective of your consent. As all the conversation is up to his/her willingness, he/she is fully entitled to ask, interrupt, skip questions and withdraw from the study any time during the interview.

**Confidentiality of the study:-** In any means the information he/she gives will not be used for other purpose beyond this study and always be kept confidential. During the interview, again based on his/her willingness, I will use an audio recorder, which means that what we talk about during the interview will be recorded. This is so that I can remember what we talked about. Your child’s name or other identification will never be mentioned in the study. The audio tape will be kept locked in a cabinet in my house and only the researcher will be allowed to listen to the audio tape. It will thereafter be destroyed.

**Benefit of the study:-** Being participant of this study by itself doesn’t have a direct benefit. However this doesn’t mean it has no benefit at all. As tried to mention in the beginning the information is helpful for improving health care services of the area. At the end of the interview session we will have tea and snack together.

**Risk of the study:-** His/her participation has no risk, in all means he/she is free of any harm and for that the researcher is responsible and accountable.

So considering the above issues I kindly request to put your response in the next page of consent form. If you have any questions you can contact me through the given address.

Thank you!

Nahom Solomon

Cell phone:- +251941246518

Email:- [nahomsolomon83@gmail.com](mailto:nahomsolomon83@gmail.com)

Addis Ababa University School of Public Health

**Parents’/Guardians’ Informed consent form**

I read/listened the above information and I understood that it is a study that doesn’t harm my child, is based only on willingness and promises confidentiality of responses. Accordingly based on my understanding, regarding my child’s participation on the study, on behalf of me without any pressure I reached on the following decision. However this doesn’t mean I enforce my child to participate. His/her participation will be assured based on his/her consent.

1. I fully agree and permit my child to participate if and only if he/she is willing
2. Do not agree

**Interviewer**

I assure that I informed and took the consent

Name ________________

Signature_______________

Date ______________­­­­­­­­­­____

## Annex-III; Amharic version of study information sheet and informed consent form

**የጥናት ተካፋዮች መረጃ**

ጤና ይስጥልኝ! እኔ ናሆም ሰለሞን በአዲስ አበባ ዩኒቨረሲቲ የሕብረተሰብ ጤና የሁለተኛ ዲግሪ ተማሪ ስሆን በአሁን ሰዓት ስለ ኤች አይ ቪ ኤድስና ተያያዥ የጤና ጉዳዮች ጥናት በማድረግ ላይ እገኛለሁ፡፡ ጥናቱ በተለይ ኤች አይ ቪ ከወላጆቻቸው የያዛቸው ወይም ከውልደታቸው ጀምሮ ኤች አይ ቪ ያለባቸውን ወጣቶች ስለሚገጥማቸው የጤና ክብካቤ ጉዳይ፤ ስለሚያሳስባቸው ጉዳይ፤ ስለሚገጥማቸው ችግሮችና ፆታዊ ባህሪና ግንኙነት ላይ ያተኮረ በጥቅሉ የህይወታቸውን ልምድ መረዳት ነው፡፡

**የጥናቱ ዓላማ፡-**ከውልደታቸው ጀምሮ ኤች አይ ቪ ያለባቸውን ወጣቶች የህይወታቸውን ልምድ ማወቅ ሆኖ የጥናቱ ውጤትም የጤናው ክብካቤ የተሻለ ይሆን ዘንድ እና የኤች አይ ቪ ስርጭትን ለመቆጣጠር ለሚደረገው ስራ አጋዥ እንዲሆን ማድረግ ነው፡፡

**የጥናቱ ሂደት፡-** ጥናቱ ዕድሜያቸው ከ15-24ዓመት ያሉትን የሚያጠቃልል ሲሆን፤ አንተ/አንቺ ከሌሎች በዚህ ጥናት የተካተትከው/የተካተትሽው እንዲሁ በአጋጣሚ እንጂ ምንም የተለየ ትኩረት ተሰጥቶ አይደለም፡፡ ጥናቱ በቃለ ምልልስ ብቻ የሚያልቅ እንጂ ምንም ዓይነት የደምም ሆነ ሌላ ምርመራ አይኖረውም፡፡ ቃለ ምልልሱ ቢያንስ 1ሰዓት ያክል የሚፈጅ ሲሆን ምናልባት የተዘለለ ሀሳብ ቢኖር እንደ አንተ/አንቺ ፈቃድ በሌላ ቀን ተመልሰን ልንነጋገር እንችል ይሆናል፡

**የጥናት ተሳታፊዎች መብት፡-**በጥናቱ ተካፋይ መሆን በአንተ/በአንቺ ፈቃድ ላይ ብቻ የተመሰረተ ነው፡፡በቃለ ምልልሱ ወቅት የፈለከውን/የፈለግሽውን ጥያቄ ማንሳት፣ ቃለ ምልልሱን ማቋረጥም ሆነ መመለስ የማትፈልገውን/የማትፈለጊውን ሀሳብ መዝለል ይቻላል፡፡

**የጥናቱ ምስጢራዊነት፡-**ይህ አንተ/አንቺ የምትሰጠው/የምትሰጪው መረጃ በማንኛውም ሁኔታ ከዚህ ጥናት ውጪ ለሆነ ጉዳይ አይውልም፤ ምሰጢራዊነቱም ሁል ጊዜ የተጠበቀ ነው፡፡ የአንተ/አንቺ በጥናቱ ተካፋይ ስትሆን/ስትሆኚ ስምህንም/ስምሽንም ሆነ ሌላ መለያህን/መለያሽን መግለፅ አይጠበቅብህም/አይጠበቅብሽም፡፡ በቃለ ምልልሳችን ወቅት የሚነሱትን ሃሳቦች ለማስታወስና የበለጠ ለመረዳት ያግዘኝ ዘንድ ማስታወሻ የምይዝ ሲሆን በተጨማሪም በመቅረፀ ድምፅ እንድቀዳ የአንትን/የአንቺን ይሁንታ በትህተና እየጠየኩኝ፤ ይህ የተያዘው ማስታወሻም ሆነ የተቀዳው ድምፅ ለዚህ ጥናት ብቻ በምስጢር የሚያዝና ከዚያ በኋላ የሚቃጠል እንደሚሆን እንዲሁም ከጥናት አድራጊው ውጪ ማንም አንደማያገኘው አረጋግጣለሁ፡፡

**የጥናቱ ጥቅም፡-** የዚህ ጥናት ተሳታፊ በመሆንህ/በመሆንሽ የተለየ ቀጥተኛ ጥቅም አይሰጥህም/አይሰጥሽም፡፡ ይህ ማለት ግን ጥናቱ ጥቅም የለውም ማለት አይደለም፤ ይልቁንም የጥናቱ ውጤት በመሰል የጤና ሁኔታ ውስጥ ያሉ ሰዎችን የጤና ክብካቤ ለማሻሻል ግብአት የሚሆንና ለበለጠ ስራ የሚረዳ ይሆናል፡፡ከዚህ ባሻገር ቃለ ምልልሳችንን እንደጨረስን ሻይ ቡና የሚኖረን ይሆናል፡፡

**የጉዳት ስጋት፡-** በዚህ ጥናት መሳተፍ ምንም ዓይነት ተፅዕኖም ሆነ ጉዳት የሌለው ሲሆን ለዚህም የጥናቱ አድራጊ ሀላፊነትና ተጠያቂነቱን ይወስዳል፡፡

እንግዲህ ከላይ ያነሳኋቸውን ሃሳቦች ከግንዛቤ በማስገባት በጥናቱ ስለመሳተፍ የደረስክበትን/የደረስሽበትን ውሳኔ ከዚህ በታች ባለው አጭር ማስታወሻ ስር እንድታመለክትልኝ/እንድታመለክቺልኝ በትህትና እየጠየኩኝ፤ ከዚህ ባለፈ መጠየቅ የምትፈልገው/የምትፈለጊው ነገር ካለ በማልንኛውም ሰዓት ከዚህ በታች በሰፈሩት አድራሻዎች መጠየቅ ይቻላል፡፡ በጣም አመሰግናለሁ!

ናሆም ሰለሞን

ስልክ +251941246518

ኢሜል [nahomsolomon83@gmail.com](mailto:nahomsolomon83@gmail.com)

በአዲስ አበባ ዩኒቨረሲቲ የሕብረተሰብ ጤና ትምህርት ክፍል

**የጥናቱ ተሳታፊዎች የስምምነት ውሳኔ መስጫ ክፍል**

እኔ ከዚህ በላይ የተገለፀውን መረጃ አንብቤ/ተነቦልኝ ሰምቼ ይህ ጥናት በእኔ ፍቃድ ላይ ብቻ የተመሰረተና በምሰጢር የሚያዝ፤እንዲሁም ምንም ጉዳትም ሆነ የተለየ ጥቅም አንደማይሰጠኝ የተረዳሁ ሲሆን በዚሁ መሰረት በጥናቱ ሥለመሳተፌ ያለምንም ግፊት በራሴው ፈቃድ የሚከተለውን ወስኛለሁ፡፡

1. እኔ በጥናቱ ለመሳተፍ ተስማምቻለሁ
2. አልስማማም

ቃለ መጠይቁን አድራጊ

እኔ ከዚህ በላይ ያለውን የስምምነት መረጃ ሰጥቼ ስምምነቱን መቀበሌን አረጋግጣለሁ

ስም ________________________

ፊርማ ______________________

ቀን ______________________

**የወላጆች/የአሳዳጊዎች የጥናት መረጃ**

ጤና ይስጥልኝ! እኔ ናሆም ሰለሞን በአዲስ አበባ ዩኒቨረሲቲ የሕብረተሰብ ጤና የሁለተኛ ዲግሪ ተማሪ ስሆን በአሁን ሰዓት ስለ ኤች አይ ቪ ኤድስና ተያያዥ የጤና ጉዳዮች ጥናት በማድረግ ላይ እገኛለሁ፡፡ ጥናቱ በተለይ ኤች አይ ቪ ከወላጆቻቸው የያዛቸው ወይም ከውልደታቸው ጀምሮ ኤች አይ ቪ ያለባቸውን ወጣቶች የተመለከተ ሲሆን እነዚህ ወጣቶች ስለሚገጥማቸው የጤና ክብካቤ ጉዳይ፤ ስለሚያሳስባቸው ጉዳይ፤ ስለሚገጥማቸው ችግሮችና ፆታዊ ባህሪና ግንኙነት ላይ ያተኮረ ሲሆን በጥቅሉ የህይወታቸውን ልምድ መረዳት ነው፡፡

**የጥናቱ ዓላማ፡-**ከውልደታቸው ጀምሮ ኤች አይ ቪ ያለባቸውን ወጣቶች የህይወታቸውን ልምድ ማወቅ ሆኖ የጥናቱ ውጤትም የጤናው ክብካቤ የተሻለ ይሆን ዘንድ እና የኤች አይ ቪ ስርጭትን ለመቆጣጠር ለሚደረገው ስራ አጋዥ እንዲሆን ማድረግ ነው፡፡

**የጥናቱ ሂደት፡-** ጥናቱ ዕድሜያቸው ከ15-24ዓመት ያሉትን የሚያጠቃልል ሲሆን፤ ከሌሎች የእርስዎ ልጅ በዚህ ጥናት የተካተተው/የተካተተችው እንዲሁ በአጋጣሚ እንጂ ምንም የተለየ ትኩረት ተሰጥቶ አይደለም፡፡ ጥናቱ በቃለ ምልልስ ብቻ የሚያልቅ እንጂ ምንም ዓይነት የደምም ሆነ ሌላ ምርመራ አይኖረውም፡፡ ቃለ ምልልሱ ቢያንስ 1ሰዓት ያክል የሚፈጅ ሲሆን ምናልባት የተዘለለ ሀሳብ ቢኖር እንደ እርስዎና ልጅዎ ፈቃድ በሌላ ቀን ተመልሰን ልንነጋገር እንችል ይሆናል፡፡

**የጥናት ተሳታፊዎች መብት፡-** በጥናቱ ተካፋይ መሆን በእርስዎ እና በልጅዎ ፈቃድ ላይ ብቻ የተመሰረተ ነው፡፡በእርግጥ እርስዎ እንኳን ፈቅደው ልጅዎ ካልፈቀደ በጥናቱ ያለመሳተፍ መብቱ የተጠበቀ ነው፡፡ በቃለ ምልልሱ ወቅት ልጅዎ የፈለገውን/የፈለገችውን ጥያቄ ማንሳት፣ ቃለ ምልልሱን ማቋረጥም ሆነ መመለስ የማይፈልገውን/የማትፈልገውን ሀሳብ መዝለል ይቻላል፡፡

**የጥናቱ ምስጢራዊነት፡-**ይህ ልጅዎ የሚሰጠኝ/የምትሰጠኝ መረጃ በማንኛውም ሁኔታ ከዚህ ጥናት ውጪ ለሆነ ጉዳይ አይውልም፤ ምስጢራዊነቱም ሁል ጊዜ የተጠበቀ ነው፡፡ልጅዎም በጥናቱ ተካፋይ ሲሆን/ስትሆን ስሙም/ስሟም ሆነ ሌላውን መለያ መግለፅ አይጠበቅም፡፡አይጠበቅብህም/አይጠበቅብሽም፡፡ በቃለ ምልልሳችን ወቅት የሚነሱትን ሃሳቦች ለማስታወስና የበለጠ ለመረዳት ያግዘኝ ዘንድ ማስታወሻ የምይዝ ሲሆን በተጨማሪም በመቅረፀ ድምፅ እንድቀዳ የእርስዎንና የልጅዎን ይሁንታ በትህተና አየጠየኩኝ፤ ይህ የተያዘው ማስታወሻም ሆነ የተቀዳው ድምፅ ለዚህ ጥናት ብቻ በምስጢር የሚያዝና ከዚያ በኋላ የሚቃጠል እንደሚሆን እንዲሁም ከጥናት አድራጊው ውጪ ማንም እንደማያገኘው አረጋግጣለሁ፡፡

**የጥናቱ ጥቅም፡-** ልጅዎ የዚህ ጥናት ተሳታፊ በመሆኑ/በመሆኗ የተለየ ቀጥተኛ ጥቅም አያገኝም/አታገኝም፡፡ ይህ ማለት ግን ጥናቱ ጥቅም የለውም ማለት አይደለም፤ ይልቁንም የጥናቱ ውጤት በመሰል የጤና ሁኔታ ውስጥ ያሉ ሰዎችን የጤና ክብካቤ ለማሻሻል ግብአት የሚሆንና ለበለጠ ስራ የሚረዳ ይሆናል፡፡ከዚህ ባሻገር ቃለ ምልልሳችንን እንደጨረስን ሻይ ቡና የሚኖረን ይሆናል፡፡

**የጉዳት ስጋት፡-** የልጅዎ በዚህ ጥናት መሳተፍ ምንም ዓይነት ተፅዕኖም ሆነ ጉዳት የሌለው ሲሆን ለዚህም የጥናቱ አድራጊ ሀላፊነትና ተጠያቂነቱን ይወስዳል፡፡

እንግዲህ ከላይ ያነሳኋቸውን ሃሳቦች ከግንዛቤ በማስገባት የልጅዎን በጥናቱ ስለመሳተፍ የደረሱበትን ውሳኔ ከዚህ በታች ባለው አጭር ማስታወሻ ስር እንድታመለክቱኝ በትህትና እየጠየኩኝ፤ ከዚህ ባለፈ መጠየቅ የሚፈልጉት ነገር ካለ በማንኛውም ሰዓት ከዚህ በታች በሰፈሩት አድራሻዎች መጠየቅ ይቻላል፡፡

በጣም አመሰግናለሁ!

ናሆም ሰለሞን

ስልክ፡- +251941246518

ኢሜል፡- [nahomsolomon83@gmail.com](mailto:nahomsolomon83@gmail.com)

በአዲስ አበባ ዩኒቨረሲቲ የሕብረተሰብ ጤና ትምህርት ክፍል

**የወላጆች/የአሳዳጊዎች የስምምነት ውሳኔ መስጫ ክፍል**

እኔ ከዚህ በላይ የተገለፀውን መረጃ አንብቤ/ተነቦልኝ ሰምቼ ይህ ጥናት በልጄ ፍቃድ ላይ ብቻ የተመሰረተና በምሰጢር የሚያዝ፤እንዲሁም ምንም ጉዳትም ሆነ የተለየ ጥቅም አንደማይሰጥ የተረዳሁ ሲሆን በዚሁ መሰረት ስለልጄ በጥናቱ መሳተፍ ያለምንም ግፊት የሚከተለውን ወስኛለሁ፤ ይህ ማለት ግን እኔ ልጄን አስገድደዋለሁ ማለት አይደለም፤ የልጄ ተሳትፎ በራሱ/በራሷ ፍቃድ ላይ ብቻ የተመሰረተ ይሆናል፡፡

1. እኔ ልጄ እስከፈቀደ/ች ድረስ በጥናቱ ላይ ይሳተፍ/ትሳተፍ ዘንድ ተስማምቻለሁ

2. አልስማማም

ቃለ መጠይቁን አድራጊ

እኔ ከዚህ በላይ ያለውን የስምምነት መረጃ ሰጥቼ ስምምነቱን መቀበሌን አረጋግጣለሁ

ስም ________________________

ፊርማ ______________________

ቀን ______________________
