# Supplementary material for: “I want to perform and succeed more than those who are HIV-seronegative” Lived experiences of youth who acquired HIV perinetally and attend Zewditu Memorial Hospital ART clinic, Addis Ababa, Ethiopia
Source: PLoS One. 2021 May 27;16(5):e0251848. doi: 10.1371/journal.pone.0251848 (PMC8158987; doi:10.1371/journal.pone.0251848)
Supplement: S2 File — (DOCX) [file pone.0251848.s005.docx]

## Annex-IV; Interview Guide

1. Socio demographic characteristics;-

- Can you tell me about yourself(**prob**:- age, education level, job, marital status, source of income, with whom are you living,
- Can you tell me about your health status(**prob**:- general, HIV status-when you became aware of it, how you knew your status, how long have you been on ART,

1. Supports of youth who acquired HIV perinatally

- Can you tell me any supportive conditions you have experienced? (**prob**:- from families, friends, neighbors, community, school, working areas, health sectors…)
- Any other supportive conditions you had and wish to be done
- With whom you prefer to discuss about your health and related condition? (**prob**:- any example experience…)

1. concerns of youth who acquired HIV perinatally

- What concerns do you have regarding to your health and related things (**prob**:- in relation to families, friends, neighbors, community, school, medias, health sectors…)
- Disclosure issues,( **prob**:- what you think about disclosing HIV status, when, how, where it should be…. )
- What do you prefer to be implemented in such area

1. Challenges of youth who acquired HIV perinatally

- Any challenges you faced (**prob**:- from your health status, families, friends, neighbors, community, school, working areas, Medias, health sectors…)
- **prob**:- Clinical appointment, ART vs school, job, social involvement
- How you cope the challenges (**prob**:- any example experiences….)

1. sexual behavior and relation of youth who acquired HIV perinatally

- What do you know about reproductive system health?
- What do you think about sexual relation (**prob**:- have you ever engaged, how? With whom, what is your future concern regarding it, what should look like your sexual relation, what you wish your sexual partner to be,)
- What do you think is your role in prevention of HIV transmission

1. any other concerns you want to share me
2. summarizing the themes
3. Thank you for your kind cooperation, I will re visit you based on your willingness for missed or untouched issues if any.

## Annex-V; Amharic version of Interview Guide

የመወያያ ነጥቦች/የቃለ ምልልሱ መነሻ ሀሳቦች

1. መግቢያ/ የጥናቱ ተሳታፊዎች ዳራ

- እድሜ
- የትምህርት ደረጃ
- እስኪ ስለኑሮ፣ ስለ ስራ እናውራ
- እስኪ ስለጤናህስ/ስለጤናሽ እናውራ
- አጠቃላይ የጤናህ/ሽ ሁኔታ
- ኤች ኤይ ቪን በተመለክተ፡-መች እንዳለብህ/ሽ አወቅህ/ሽ፣ እንዴትስ አወቅህ/ሽ፣ መድሀኒት መጠቀም ከጀመርክ/ሽ ስንት ጊዜ ሆነህ/ሽ

1. ኤች አይ ቪ ከወላጆቻቸው የያዛቸው ወጣቶች ያሏቸው ደጋፊ ነገሮች

- እስኪ ያየሀቸው/ያየሻቸው ደጋፊ ነገሮችን ንገረኝ/ንገሪኝ(ለምሳሌ ከቤተሰብ፣ ከትምህርት ቤት፣ ከጎርቤት፣ከማህረሰቡ፣ ከመገናኛ ብዙሀን…..
- ሌላ ተጨማሪ ከገጠሙህ/ሽ እና እንዲህ ቢሆን የምትለው/የምትዪው…….
- ከማን ጋር ነው በጤናህ/ሽ ጉዳይ የምተነጋገረው/ሪው….ከዚህ ጋር ተያይዞ የገጠመህ/ሽ ነገር ካለ

1. ኤች አይ ቪ ከወላጆቻቸው የያዛቸው ወጣቶች ያሉባቸው አሳሳቢ ጉዳዮች

- ከጤናህ/ሽ ጋር በተያያዘ ምን ምን ነገሮች ናቸው የሚያሳስቡህ/ሽ(ለምሳሌ ከቤተሰብ፣ ከትምህርት ቤት፣ ከጎርቤት፣ከማህረሰቡ፣ ከመገናኛ ብዙሀን ጋር በተያያዘ…..
- የራስህን/ሽን የጤና ሁኔታ ከመግለፅ እንፃር

1. ኤች አይ ቪ ከወላጆቻቸው የያዛቸው ወጣቶች ያሉባቸው ተግዳሮቶች/ፈታኝ ነገሮች

- የገጠሙህ/ሽ አስቸጋሪ/ፈታኝ ነገሮች (ለምሳሌ ከቤተሰብ፣ ከትምህርት ቤት፣ከሀኪም ቤት፣ ከጎርቤት፣ከማህረሰቡ፣ ከመገናኛ ብዙሀን ጋር በተያያዘ…..
- እስኪ ከመድሀኒቱ አና ህክምናው ጋርስ በተያያዘ(ለምሳሌ የሀኪም ቤት ቀጠሮ፣ የመድሀኒት መውሰጃ ሰዓትና ጥምህርት……..
- አስቸጋሪ/ፈታኝ ነገሮችን እንዴት ነው የምታልፈው/ፊው

1. ኤች አይ ቪ ከወላጆቻቸው የያዛቸው ወጣቶች ያሉባቸው ያላቸው ፆታዊ ባህሪና ፆታዊ ግንኙነት ልምድ

- ስለ ስነ ተዋልዶ ጤናና ስነ ፆታ የምታውቀውን/የምታውቂውን ነገር ንገረኝ/ንገሪኝ እስኪ
- ፆታዊ ባህሪና ግንኙነትን በተመለከተ ያለህ/ሽ ሃሳብና ልምድ፣ (ከዚህ በፊት ግንኙት ነበረህ/ነበረሽ፤ ከሆነ እንዴት ነበር፤ ወደ ፊትስ ምን ዓይነት ግንኙነት እንዲኖርህ/ሽ ታስባለህ/ሽ
- ኤች አይ ቪን ከመከላከል አንፃር ያንተስ/ያንቺስ ድርሻ ምን መሆን አለበት ብለሽ ታስባለህ/ታስቢያለሽ

1. ሌላ ተጨማሪ ማንሳት የምትፈልገው/ጊው ነገር ካለ
2. የተነሱትን ጭብጥ ጉዳዮች በመከለስ ማጠናቀቅ
3. ስለ ቀና ትብብርህ/ሽ በጣም አመሰግናልሁ፡፡ ምናልባት የተረሳ ነገር ካለ እና ፈቃድህ/ሽ ከሆነ ሌላ ጊዜ መልሼ ላናግርህ/ሽ እችላለሁ፡፡
